# Left Ventricular and Lung Perforation Caused by a Pacemaker Lead Requiring Emergency Surgical Repair in a Nonagenarian

**DOI:** 10.7759/cureus.107215

**Published:** 2026-04-17

**Authors:** Nikoleta Stanitsa, Viktor Panagiotakopoulos, Emmanouil Tempelis, Dimitris Tzalas, Evangelos Papageorgiou, Panagiotis Dedeilias

**Affiliations:** 1 Cardiothoracic Surgery, Evangelismos General Hospital, Athens, GRC; 2 Cardiac Surgery, Evangelismos General Hospital, Athens, GRC; 3 Cardiology, Evangelismos General Hospital, Athens, GRC; 4 Anesthesiology, Evangelismos General Hospital, Athens, GRC

**Keywords:** delayed complication, emergency surgery, epicardial pacing, left ventricular perforation, pacemaker lead perforation, pulmonary involvement

## Abstract

Cardiac perforation is an uncommon but potentially life-threatening complication of permanent pacemaker implantation. Delayed perforation involving extracardiac structures is rare; left ventricular free-wall perforation with pulmonary extension is exceptionally uncommon and may be diagnostically challenging, especially when pericardial effusion is absent. A 91-year-old man residing on a Greek island developed delayed perforation of the presumed interventricular septal/paraseptal course with exit through the left ventricular free wall and extension toward the left lung after dual-chamber pacemaker implantation for symptomatic high-grade atrioventricular conduction disease/bradyarrhythmia. Initial computed tomography demonstrated a small left pneumothorax and localized pulmonary hematoma without pericardial effusion. The patient subsequently deteriorated with severe bradycardia and pacing instability, requiring pharmacologic chronotropic/hemodynamic support and urgent air transfer to a tertiary cardiac surgical center. Emergency surgical repair via full median sternotomy was performed without cardiopulmonary bypass. The ventricular lead was withdrawn under direct vision from the left ventricular free-wall perforation and then transected under controlled traction. The left ventricular perforation was repaired with polypropylene sutures. Temporary epicardial atrial and ventricular pacing wires were placed to ensure reliable postoperative rhythm support and hemodynamic stabilization. After clinical stabilization, a definitive permanent pacemaker was implanted using the retained atrial lead and a newly placed ventricular lead to restore dual-chamber pacing. There were no clinical signs of device-related or systemic infection before reimplantation. Recovery was uneventful, and at three-month follow-up, the patient remained asymptomatic with normal device function.

This case aims to improve recognition of delayed pacemaker lead perforation as a potential cause of clinical deterioration, even in the absence of pericardial effusion. It also underscores the importance of surgical treatment and the use of temporary epicardial pacing when the perforating lead involves extracardiac structures. Finally, it highlights the need for early cross-sectional imaging and a multidisciplinary approach to decision-making, particularly in very elderly patients.

## Introduction

Permanent pacemaker implantation is routinely performed with a favorable safety profile; however, clinically meaningful complications may occur and can be associated with substantial morbidity and mortality. Lead perforation is an infrequent but potentially life-threatening adverse event; in a large single-center cohort, symptomatic perforation presenting with significant pericardial effusion occurred in 1.2% of implants, while the overall perforation rate reported across studies is generally <1% for pacemakers [1].

Lead perforation may be acute, subacute, or delayed, with delayed perforation commonly defined as occurring beyond 24 hours after implantation. Delayed cases may present with atypical symptoms or subtle clinical findings, contributing to diagnostic delay [2]. Most reported perforations involve the right ventricle, while left ventricular perforation is exceptionally rare. Extension into extracardiac structures, particularly the pleura or lung, further complicates management, frequently necessitating surgical intervention [3].

Older adults may be particularly vulnerable due to myocardial thinning, reduced tissue elasticity, and a higher prevalence of comorbidities or concurrent anticoagulation. We report a rare case of delayed transvenous pacemaker lead perforation with a likely septal trajectory, left ventricular free-wall exit, and pulmonary involvement in a nonagenarian patient, successfully managed with emergency surgical repair and a staged pacing strategy.

## Case presentation

A 91-year-old man residing on a Greek island underwent dual-chamber atrioventricular pacemaker implantation via the subclavian vein for symptomatic high-grade atrioventricular conduction disease/bradyarrhythmia. According to the available transferred documentation, the exact commercial model of the pulse generator and the exact model numbers of the leads used at the index implantation were not retrievable. The ventricular lead was understood to be an active-fixation transvenous lead; however, complete manufacturer-specific technical details were unavailable from the outside institution. The patient was discharged from the referring hospital on the first post-implantation day in stable condition. Four days after implantation, he developed worsening fatigue and presented to the local hospital for evaluation.

**Figure 1:**
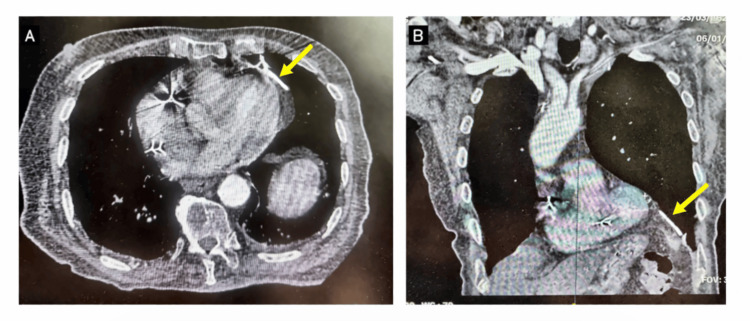
Computed tomography demonstrating pacemaker lead perforation with likely septal/paraseptal course, left ventricular free-wall exit, and extracardiac extension (A) Axial contrast-enhanced chest CT showing the pacemaker ventricular lead beyond the expected intracardiac course, with perforation through the left ventricular free wall and extension toward the left hemithorax. (B) Coronal CT reconstruction further demonstrates extracardiac extension of the lead toward the left pleural space and adjacent lung parenchyma. Although the original chest radiographs and additional lung-window CT reconstructions were unavailable for submission, the outside radiologic evaluation described a small associated left pneumothorax and focal pulmonary hematoma.

Computed tomography performed at the local facility demonstrated perforation of the pacemaker ventricular lead along a course most consistent with septal/paraseptal traversal and exit through the left ventricular free wall, with extension toward the left lung parenchyma (Figure 1). Imaging further revealed a small left-sided pneumothorax and a localized pulmonary hematoma. Notably, there was no pericardial effusion and no radiologic evidence of cardiac tamponade.

Over the ensuing period, the patient’s condition deteriorated clinically with marked weakness, symptomatic severe bradycardia (ventricular rate approximately 38 bpm), pacing instability, and hemodynamic compromise. Pharmacologic chronotropic/hemodynamic support was initiated at the referring hospital before transfer; however, the exact drug regimen and infusion doses were not completely available in the transferred documentation. Given the complexity of the complication and the absence of local cardiac surgical capability, the patient was urgently transferred by air ambulance to a tertiary referral center for definitive management.

Operative management

**Figure 2:**
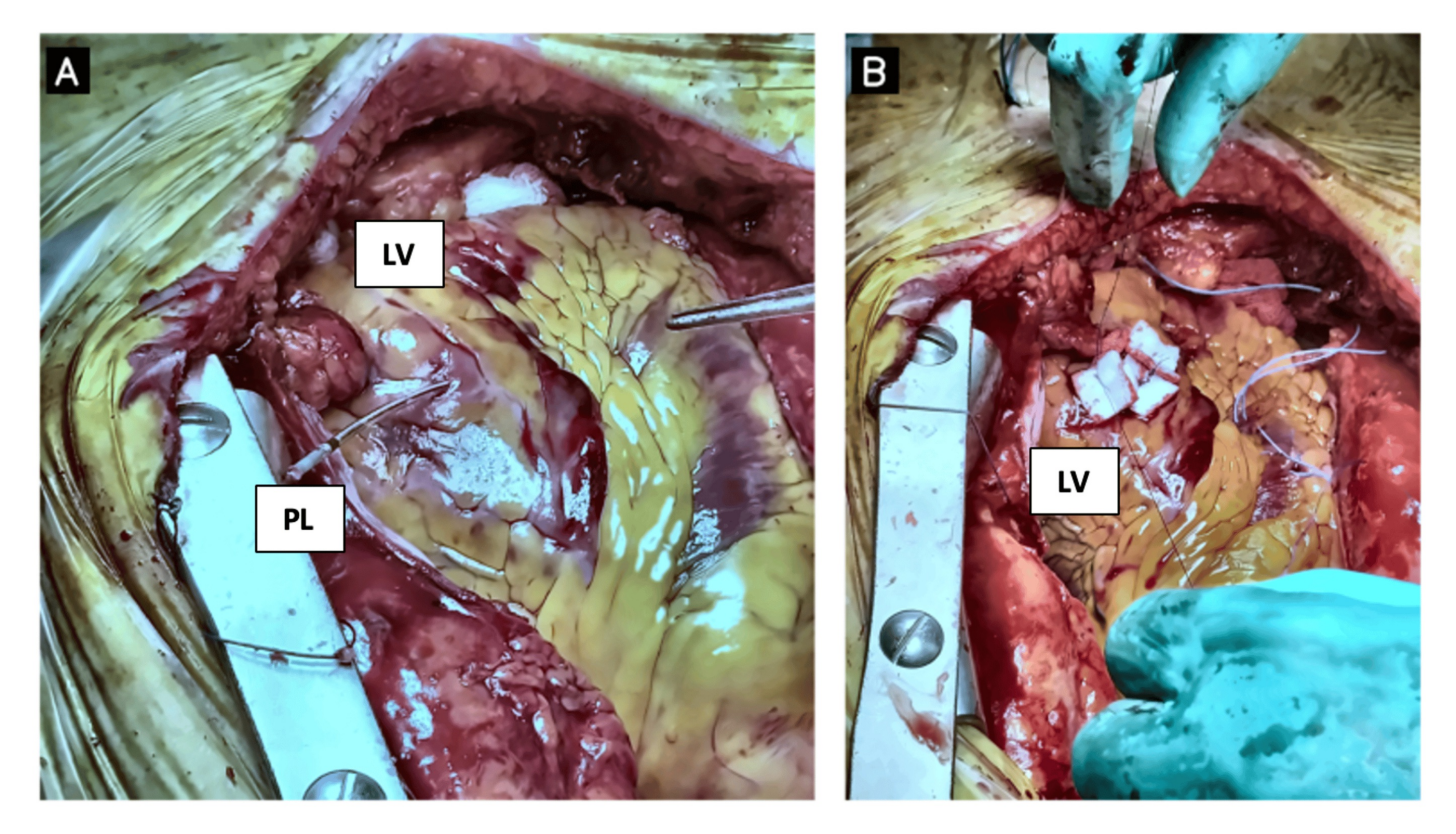
Intraoperative findings and repair of left ventricular lead perforation (A) Intraoperative view demonstrating the ventricular pacing lead protruding through the anterior left ventricular free wall adjacent to the course of the LAD, supporting left ventricular perforation. (B) After placement of felt-pledgeted 4-0 polypropylene sutures and lead withdrawal/transection, the repaired perforation site is shown with temporary epicardial pacing wires in situ for postoperative rhythm support. LV: left ventricle, PL: pacing lead.

Emergency surgery was performed under general anesthesia via full median sternotomy. Intraoperative findings confirmed perforation of the left ventricular free wall by the pacemaker lead with extension toward the adjacent left lung. The ventricular entry point was identified on the anterior surface of the left ventricle in proximity to the left anterior descending (LAD) coronary artery, supporting left ventricular rather than right ventricular free-wall involvement (Figure 2A). There was no pericardial effusion and no evidence of tamponade. Two temporary epicardial pacing wires-one atrial and one ventricular-were placed at the beginning of the procedure to ensure immediate rhythm support and to minimize reliance on transvenous pacing in the setting of recent ventricular injury. The left ventricular perforation site was then identified. Two 4-0 polypropylene (Prolene) pledgeted (felt-reinforced) sutures were placed circumferentially around the perforation without initially tying them. Under direct vision, the malpositioned ventricular lead was carefully retracted from the defect and transected under controlled traction, after which the pre-positioned pledgeted sutures were tied to close the perforation (Figure 2B). Meticulous hemostasis was achieved.

The patient was transferred to the intensive care unit for close hemodynamic and rhythm monitoring and remained stable during the first 24 hours. After clinical improvement, he was transferred to the cardiac surgery ward. Forty-eight hours later, definitive permanent pacing was re-established: the malfunctioning/malpositioned ventricular lead and generator were removed, the atrial lead was retained in situ, and a new ventricular lead was implanted with correct intracardiac positioning, restoring dual-chamber pacing. Before reimplantation, there were no clinical signs of local pocket infection, bloodstream infection, fever, or other evidence suggesting device-related infection.

The remainder of the hospital course was uneventful. After removal of the surgical drain, the patient demonstrated progressive recovery and was discharged home on postoperative day six. At three-month follow-up, he remained clinically stable with normal device function and no evidence of recurrent cardiac or pulmonary complications.

## Discussion

Pacemaker lead perforation is rare but clinically consequential, and delayed presentations can be particularly challenging because symptoms may be nonspecific and standard bedside studies may be unrevealing [4]. Importantly, the absence of pericardial effusion does not exclude myocardial perforation, especially when the lead traverses myocardium and decompresses into adjacent extracardiac spaces rather than into the pericardial cavity [5]. This feature can falsely reassure clinicians and delay the escalation of diagnostic imaging and definitive treatment.

Multiple risk factors have been associated with lead perforation, including advanced age, female sex, low body mass index, active-fixation leads, steroid therapy, and anticoagulation/antiplatelet therapy. In very elderly patients, myocardial thinning and increased tissue fragility may predispose to delayed transmural migration, particularly with repetitive cardiac motion and micro-trauma at the lead-myocardial interface [6].

Although most perforations occur in the right ventricle, left ventricular perforation is exceptionally rare because ventricular leads are routinely positioned in the right ventricle. In the present case, the available CT and operative findings suggest that the lead course was more compatible with septal or paraseptal positioning, with subsequent progression through the interventricular septal region and exit via the anterior left ventricular free wall, rather than a true right ventricular apical perforation. This interpretation is anatomically plausible and may explain how the lead reached the left ventricle before exiting extracardially. Proposed mechanisms in reported cases include inadvertent septal penetration during implantation with subsequent migration, or progressive transmural erosion under mechanical stress [5]. When extracardiac extension occurs, pulmonary complications such as pneumothorax, pulmonary hemorrhage/hematoma, pleural effusion, or chest pain may develop and increase morbidity [7]. The combination of left ventricular perforation and pulmonary involvement, therefore, represents a particularly high-risk phenotype requiring a low threshold for urgent intervention.

While chest radiography and echocardiography are often first-line tests in suspected lead complications, both can be nondiagnostic in delayed perforation, particularly when there is no pericardial effusion or when the lead tip has migrated beyond the expected cardiac silhouette without overt radiographic change. In this context, computed tomography is highly valuable: in a comparative study, diagnostic accuracy was 73.1% for chest radiography and 82.7% for transthoracic echocardiography, compared with 98.1% for ECG-gated contrast-enhanced cardiac CT, which can directly delineate lead trajectory, confirm extracardiac extension, and identify associated thoracic complications such as pneumothorax or parenchymal hematoma [8]. In the present case, CT rapidly established the diagnosis and, critically, demonstrated pulmonary involvement without tamponade, informing the need for urgent surgical planning despite the absence of pericardial effusion. We acknowledge that additional chest radiographs and lung-window CT images would have strengthened the visual documentation of this case; however, these were not retrievable from the original referring-hospital archive for inclusion in the present report.

Management ranges from observation to percutaneous lead extraction or surgical repair, depending on symptoms, hemodynamics, pacing function, and the presence of extracardiac injury [9]. Asymptomatic or incidental micro-perforations may be monitored in selected cases. However, expert consensus and accumulated clinical experience support surgical management when there is hemodynamic compromise, unstable pacing, clear ventricular free-wall perforation, or injury to extracardiac structures [10].

In our patient, several factors favored an aggressive approach: (i) left ventricular free-wall perforation, (ii) pulmonary extension with pneumothorax/hematoma, (iii) severe bradycardia and pacing instability, and (iv) extreme age with limited physiologic reserve. Surgical exposure allowed controlled lead withdrawal under direct vision, definitive repair of the ventricular defect, and immediate establishment of reliable rhythm support.

Temporary epicardial atrial and ventricular pacing wires provided a safe “bridge” strategy after ventricular repair. This approach is particularly useful when immediate reliance on the compromised transvenous system is unsafe or when transvenous manipulation could jeopardize a fresh ventricular repair site. In addition, epicardial pacing helps stabilize rhythm and hemodynamics while clinicians reassess device function and determine the safest timing for definitive system revision or reimplantation.

In the present case, definitive device revision was performed after stabilization: the generator and malpositioned ventricular lead were removed, the atrial lead was retained in situ, and a new ventricular lead was implanted with correct intracardiac positioning to restore dual-chamber pacing. This staged approach aligns with a safety-first strategy often adopted in complex perforations with extracardiac injury, where immediate transvenous reintervention may increase risk. Equally important, reimplantation was undertaken only after clinical reassessment confirmed the absence of signs of local or systemic infection and after the patient had recovered uneventfully from the median sternotomy.

This case underscores that delayed pacemaker lead perforation can present with deterioration even in the absence of pericardial effusion. Early CT imaging and multidisciplinary coordination are essential, and advanced age alone should not preclude definitive surgical management when the clinical scenario mandates it.

## Conclusions

Delayed left ventricular and pulmonary perforation following pacemaker implantation is a rare but life-threatening complication. Early recognition, prompt cross-sectional imaging, urgent transfer to a specialized cardiac surgical center, and decisive multidisciplinary management are essential. Surgical repair with temporary epicardial pacing followed by definitive pacemaker system revision can be a safe and effective strategy, even in extremely elderly patients. This case also suggests that when the lead trajectory is not anatomically compatible with a simple right ventricular apical perforation, a septal or paraseptal course should be considered during imaging review and operative planning.
